# Practical Points on Porcelain Fillings

**Published:** 1898-08

**Authors:** W. A. Capon

**Affiliations:** Philadelphia, Pa.


AMERICAN JOURNAL

—OF—

DENTAL SCIENCE.

VOL. XXXII. BALTIMORE, AUGUST, 1898. No. 4.

### Selections.

ARTICLE I.

### PRACTICAL POINTS ON PORCELAIN FILLINGS.

W. A. CAPON, D. D. S., PHILADELPHIA, PA.

Mr. President and fellow-members of the Alumni Society of the Philadelphia Dental College:—The title of my paper, "Practical Points on Porcelain Fillings," seems to me very appropriate on this particular occasion from the fact that this class of work first received its impetus in this community within the walls of the Philadelphia Dental College, in the year 1889 I was then a senior student and very much impressed with the possibilities of this work, and extremely anxious to practice and perfect myself in this new branch of dentistry. My preceptor, knowing my inclinations and tendency to natural shading and small manipulative work, urged on me the importance of securing the practice afforded by a college clinic. Through his influence with Doctor Land, of Detroit, the inventor of the furnace and perfector of the work, I secured a furnace. I then interviewed some of the members of the faculty and although skeptical and somewhat curious, and no doubt amused at my enthusiasm, kindly granted me permission to tap a gas pipe in the operating room. From that time till the term expired, the noise of that little furnace could be heard almost every day, and I made porcelain inlays till my heart was content. The boys called me a "porcelain crank," but I industriously continued to make fillings for all kinds of places, and crowns never thought of before or since; and yet, after nearly nine years, I am proud and pleased to know that much of that work was successful and is doing excellent service to day, and many of my college patients are my staunchest friends and firm adherents of porcelain fillings. It is very true that some of it failed, and it would be very remarkable if it had not, when one considers the disadvantages that had to be overcome and my small knowledge of what I was doing, or what might be expected of it. Years afterward, I look back on that short college term with a considerable degree of satisfaction, for it enabled me to start a foundation that I was privileged to continue after a year of special instruction in this particular branch.

Porcelain fillings, inlays or sections form a part of dentistry distinctly its own and in no way antagonizing the other portions of operative work, for all practicing dentists have cases where teeth are only saved by continual patching and refilling with cement or gutta-percha, putting off the evil day when the tooth will have to be devitalized and crowned. In a case such as mentioned, and not at all exaggerated, a knowledge of porcelain fillings, and the means of making them, might have saved the tooth and probably the reputation of the dentist. Not only is this work distinct because it fills a long desired want, but its character is decidedly different from other work, and must be treated with caution and practiced with extreme conservatism. Added to this, one must have a good bump of location and a natural tendency to shading and manipulative dexterity. Those fortunate in having these combinations, or a portion of them, and the perseverance to acquire the rest, can be safe in feeling that practice will make him a porcelain worker, or, a preferable term in my estimation, an art dentist. Of course we all know that a man or woman may be an artistic dentist in many other ways, in the laboratory as well as in the operating room. but the most artistic gold filling must be secondary to a well adapted, correctly shaded and properly contoured porcelain filling. The former requiring close and continued application over a tied-up and, in many cases, nervous patient, while the picture of the latter is much more pleasant to behold, and decidedly more acceptable to the patient, and the result must be artistic because you have not only restored the original form, but the shade also, and approximated the texture of the tooth. I have advised caution and conservatism because experience has taught me their value, and such practice will insure reasonable success. There is a certain fascination about this work that, if you are not well guarded, trouble will be met with. It seems to be a tendency of a beginner in any specific branch, whether it be porcelain, making bridges or gold caps, to overdo it, and he goes gloriously on, wondering why everybody does not come to him for their dentistry till, to use a term of the day, he gets a puncture in the shape of some "thank you" work, and then, if he is wise, the first entry on the experience page will be made. I know dozens of dentists who have in the last few years taken up porcelain work, and after a little while given it up, completely disgusted and convinced that there is nothing in it, and express surprise that I am still an adherent and meet with success. Some of these parties I have put on the right track, while others were content to leave it alone.

To have you understand more readily what I consider safe practice, I will, for your guidance, divide the cavities in three grades, and call them suitable, doubtful and those to be avoided. To be more explicit I will show at the conclusion of this paper a few sketches of teeth and their cavities in the order named. Among the most suitable places for porcelain inlays, fillings or sections I will name the cervical margins on the labial and buccal surfaces of all teeth from bicuspids to bicuspids, upper and lower, but particularly the upper, proximate cavities, corner and cross sections of the oral teeth; under the second head, or doubtful, place proximate pin-head cavities, proximal margins and distal proximate surface of bicuspids, and in the last case, or those to be avoided, place all molar cavities. The coronal surface of bicuspids, very small corners on incisors, also the abraded edges of the same teeth. There is one exception in the last list that can be safely placed among those of the best, and that is the mesial proximate surface of first molar, simple or compound cavity, where space has been made by the loss of second bicuspid. This allowing good access for impression and withdrawal of matrix. The cavities in the preferred list are all accessible or can be made so by separation. The second class may be reduced by the dexterity and experience of the operator if so desired, but as a rule, pin-hole fillings are difficult to handle, and not much to be gained by using them; but of course in this work, as in all other, you must be governed by circumstances. Those in the last list are so placed because of the impossibility of getting a good impression, and the great pressure brought to bear on a brittle substance on the grinding surface of molars and bicuspids.

The preparation of a cavity for the reception of porcelain is similar to that which is intended for gold or plastic work, excepting the edges, which must be made square to allow of a better joint A slight undercut is all that is necessary; otherwise the impression cannot be withdrawn freely. Plenty of space is imperative if filling between the teeth, for, unlike other fillings, they are finished before inserting, and being a brittle, unyielding substance, there is danger of breaking if forced where the space is insufficient.

The use of a Perry or ivory separator will assist very much in many instances. The platinum foil must be thoroughly annealed and will resemble ordinary tin foil that you are all familiar with. The metal is gently forced in the cavity with a small rubber tip, and then use an amalgam burnisher. The uninitiated are always dismayed at the hole that is invariably made through the foil, but that cannot be helped and is no objection.

The most important part of the whole procedure is the second burnishing after the first baking. It is then the edges are forced in place and the true fit determined. Carelessness, or an oversight at this time, will likely prove disastrous to the filling; therefore I must impress on you the importance of the second burnishing. When the filling is finished, remove the platinum and cut a groove on at least two sides with a rubber corundum or diamond disk, and cement in position. You may have excellent joints and a perfect shade, but the cement may spoil it all, especially those thin margin fillings were shade is the great requisit. Try to use a shade of cement as near the tooth as possible, and mix thin, otherwise it may pack and keep filling from going in place. If this should occur, withdraw it quickly, and commence again; then dry with hot air and cover with parafin wax, rubber or sandarac varnish. Leave the dressing of the edges till thoroughly hardened, and after a time you will be pleased with the apparent blending of shade in tooth and filling that was temporarily changed at the time of operation.

There are many discouraging things in the practice of this work; for instance, you may loose the filling when all ready for insertion; or it may drop and get stept on; or the fit may be wretched, or the color away off. But just say to yourself, others have done this satisfactorily, and I will do it also; and if you succeed, your best friend will be your patient. Your brother dentists may laugh, and say they are no good' anyway, for they will not last. If you feel that you are on the right track, do not mind these little discouragements and delays, for I have been through it all many, many times, and I am still pegging away, and now have hundreds of cases to back my assertion and prove success. I have a filling in my right lateral, inserted by my brother nine years ago, and recent inspection shows it to be all right, and I have seen some put in by Doctor Land twelve years ago and apparantly as good now as when first made. Bear in mind that this class of work is not easy, and cannot be hurried over, for in no other branch of dentistry is that old saying more applicable. "First do it well, and then do it easily."—*Dental Brief.*

